# Evaluation of seminal plasma levels of vaspin and visfatin in infertile males with elevated sperm DNA fragmentation index: a comparative study

**DOI:** 10.1186/s12610-024-00234-1

**Published:** 2024-11-05

**Authors:** Medhat Kamel Amer, Neveen Ahmed Youssef, Sameh Fayek GamalEl Din, Nada Gamal Saied, Merna Ayman Ahmed, Ahmad Raef, Ahmed Ragab

**Affiliations:** 1 Department of Andrology, KasrAlainy Faculty of Medicine, Cairo University, Sexology, and STIs, Cairo, Egypt; 2 Adam International Hospital, Giza, Egypt; 3 Department of Clinical and Chemical Pathology, Faculty of Medicine, Beni-Suef University, Beni-Suef, Egypt; 4 Egyptian Ministry of Health and Population, Cairo, Egypt; 5 Department of Andrology, Sexology, and STIs, Faculty of Medicine, Beni-Suef University, Beni-Suef, Egypt; 6 Department of Andrology and STDs Kasr Al-Ainy, Faculty of Medicine, Cairo University, Al-Saray Street, El Manial, Cairo, 11956 Egypt

**Keywords:** Male infertility, Sperm DNA fragmentation index, Seminal plasma vaspin, Seminal visfatin, Sperm chromatin dispersion assay, Infertilité masculine, Indice de fragmentation de l'ADN spermatique, Vaspine du plasma séminal, Visfatine du plasma séminal, Test de dispersion de la chromatine des spermatozoïdes

## Abstract

**Background:**

Sperm DNA fragmentation (SDF) can significantly impact male fertility, especially in cases where there is a substantial level of DNA damage. We aimed in the current study to assess seminal plasma (SP) levels of vaspin and visfatin in infertile men with an elevated SDF index (SDFI ≥ 30%) compared to infertile males with a normal SDFI (SDFI < 30%).

**Results:**

Groups with good and medium DNA integrity exhibited significantly higher total motile sperm count and sperm motility in comparison to the group with poor DNA integrity. Significant negative correlations were noticed between SDF index (SDFI) and numerous semen parameters. Similarly, a significant negative correlation was observed between SDFI and SP vaspin. On the other hand, a significant positive correlation was found between SDFI and abnormal forms percentage. A statistically significant negative correlation was identified SP vaspin with age (*r* = -0.305, *P* = 0.006) and infertility duration (*r* = -0.263, *P* = 0.019). Statistically significant negative correlation was also identified between SP visfatin and abnormal forms percentage (*r* = -0.239, *P* = 0.034). The receiver operating characterisitic curve for predicting poor DNA integrity (SDFI ≥ 30%) revealed fair discriminative power for SP vaspin, with a cutoff value of < 0.55 ng/ml. It demonstrated a sensitivity of 58.8% and a specificity of 64.5% (area under the cureve (AUC) 0.685, *p* = 0.008). Meanwhile, SP visfatin had little discriminative power (AUC 0.562, *p* = 0.408). Finally, the results of a linear regression analysis indicated that sperm motility and SP vaspin were significant independent predictors of poor DNA integrity (SDFI ≥ 30%). The analysis was done with a 95% confidence interval and showed upper and lower bounds of -0.302 and -0.623, and -1.362 and -16.101, *p* < 0.001, *p* = 0.021, respectively.

**Conclusion:**

SP Level of vaspin had shown promise as potential biomarkers for sperm DNA integrity. However, vaspin appeared to have greater specificity than visfatin in this point. Future studies are required to validate these findings, evaluate the role of SP vaspin in maintaining sperm DNA integrity, and investigate the potential relationship between SP adipocytokines and other clinical-demographic variables.

## Résumé

**Contexte:**

La fragmentation de l'ADN des spermatozoïdes (SDF) peut avoir un impact significatif sur la fertilité masculine. Dans cette étude, nous avons cherché à évaluer les niveaux de vaspine et de visfatine dans le plasma séminal (PS) chez les hommes infertiles présentant un indice SDF élevé (SDFI≥30%) par rapport aux hommes infertiles présentant un SDFI normal (SDFI <30%).

**Résultats:**

Les patients dont l'intégrité de l'ADN spermatique était bonne ou moyenne présentaient un nombre total de spermatozoïdes mobiles et une mobilité significativement plus élevés par rapport au groupe dont l'intégrité de l'ADN était mauvaise. Des corrélations négatives significatives ont été observées entre l'indice SDF (SDFI) et plusieurs paramètres du sperme. De même, une corrélation négative significative a été observée entre le SDFI et le niveau de vaspin dans le PS. Par ailleurs, une corrélation positive significative a été trouvée entre le SDFI et le pourcentage de formes anormales. Une corrélation négative statistiquement significative a été identifiée entre le niveau de vaspin dans le PS et l'âge (r= -0,305, p=0,006) et la durée de l'infertilité (*r*= -0,263, *p*=0,019). Une corrélation négative statistiquement significative a également été identifiée entre le niveau de visfatine dans le PS et le pourcentage de formes anormales (*r*= -0,239, *P*=0,034). La courbe ROC prédit une mauvaise intégrité de l’ADN spermatique (sensibilité 58,8%, spécificité 64,5%, aire sous la courbe 0,685, *p*=0.008) lorsque la teneur en vaspine dans le PS est inférieure à 0,55 ng/ml.

En revanche, la teneur du PS en visfatine s’est avérée avoir un faible pouvoir discriminant (AUC 0,562, *p* = 0,408). Enfin, les résultats d'une analyse de régression linéaire ont indiqué que la mobilité des spermatozoïdes et le niveau de  vaspine dans le PS étaient des prédicteurs indépendants significatifs d'une mauvaise intégrité (SDFI ≥ 30 %) de l'ADN spermatique. L'analyse a été réalisée avec un intervalle de confiance de 95 % et a montré des limites supérieures et inférieures de -0,302 et -0,623, et de -1,362 et -16,101, *p*<0,001, *p*=0,021, respectivement.

**Conclusion:**

Les niveaux de vaspin dans le PS se sont révélés prometteurs en tant que biomarqueurs potentiels de l'intégrité de l'ADN des spermatozoïdes. Toutefois, la vaspine semble avoir une plus grande spécificité que la visfatine sur ce point. De futures études sont nécessaires pour valider ces résultats, évaluer le rôle de la vaspine SP dans le maintien de l'intégrité de l'ADN des spermatozoïdes et étudier la relation potentielle entre les adipocytokines du plasma séminal et d'autres variables cliniques et démographiques.

## Introduction

Sperm DNA fragmentation (SDF) can significantly impact male fertility, especially in cases where there is a substantial level of DNA damage. Furthermore, even low levels of oxidative stress (OS) can still have detrimental effects on various aspects of sperm function by impairing motility, acrosomal exocytosis, and sperm-oocyte fusion [1]. SDF testing could be fundamental for daignosing infertility and predicting assisted reproductive technology success [2]. Although, numerous techniques for SDF testing are available, yet, no single dedicated test has been recognized to show reliability in predicting the chance of pregnancy in the era of medically assisted reproduction [3]. The sperm chromatin dispersion (SCD) test is a reliable method for evaluating SDF. It is recognised for its ease, quickness, and precision [4]. Adipose tissue is a vital endocrine organ that secretes cytokines and hormones. It is also known to have an impact on male fertility [5]. Obesity can lead to a reduction in semen parameters through increased scrotal temperature as well as systemic inflammation and OS initiated by adipose tissue-derived molecules [6]. Adipokines may play a role in linking obesity to infertility [7] and could potentially serve as biomarkers for male infertility [8]. Vaspin is a recently identified adipocytokine expressed in reproductive and adipose tissues, with limited research on its impact on male fertility [9]. It is a glyco-protein that belongs to serine protease inhibitor family known as visceral adipose-specific serpin or serpin A12 [10]. Also, it acts as an anti-inflammatory adipokine with insulin-sensitizing and appetite-suppressing effects [11, 12]. Remarkably, it’s level was reported to be significantly higher in seminal plasma (SP) compared to serum. Nevertheless, its serum level was linked to impaired semen parameters in animal models [9, 13]. Visfatin, or nicotinamide phospho-ribosyl transferase, is a newly identified adipokine whose SP level is also 100 times greater than that of serum [14].

Visfatin regulates nicotinamide adenine dinucleotide (NAD) levels, an essential co-enzyme in cellular metabolism, with a significant effect on Sertoli cells. It’s reduced form, NAD + hydrogen (NADH), is implicated in the capacitation of human spermatozoa, a critical event for male fertilization potential [15]. Throughout the past ten years, a lot of research has been conducted on relationship between circulating adipokines and female infertility. Although certain adipokines, such as visfatin, were found in SP, yet, research on male side had been lacking [16]. We aimed in the current study to assess SP levels of vaspin and visfatin in infertile men with an elevated SDF index (SDFI) ≥ 30% compared to infertile males with a normal SDFI (< 30%) in order to ascertain whether these adipokines could serve as potential biomarkers for evaluating the integrity of sperm DNA.

### Patients and methods

The current prospective comparative study was conducted from May 2022 to September 2023 and included ninety infertile males. Individuals were enrolled in the study after being referred to the semen lab of a specialised IVF centre (Adam International Hospital, Giza, Egypt) for SDF evaluation. Eligible participants were requested to sign an informed consent prior to involvement in this study, as mandated by the Research Ethical Committee of Beni-Suef Faculty of Medicine and in accordance with Helsinki Declaration 2013 [17]. Ethical approval is registered under number FMBSUREC/05072022. Participants were then split into three categories based on their SDFI values: those with SDFI 0–15% (group I, normal SDFI), 15% < SDFI < 30% (group II, average SDFI), and SDFI ≥ 30% (group III, high SDFI).

### Inclusion criteria

Infertile males over the age of 20, displaying either primary or secondary infertility, along with abnormalities in one or more of the following parameters including sperms motility, sperms concentration, or sperms morphology were enrolled.

### Exclusion criteria

Men who had sperms concentration of < 5 million/ml, as well as those with cryptozoospermia, azoospermia, or leukocytospermia (leucocytic count > 1 million/ml), were excluded. Patients who were morbidly obese (body mass index (BMI) ≥ 35 kg/m^2^) or had received anti-oxidant therapy within the previous three months were also excluded.

All patients were subjected to the following:

Medical and operative histories were obtained. General and genital examinations as well as measurement of BMI and signs of hypogonadism were done for all participants. A history of antioxidant and/or antibiotic prescription use was also verified.

#### Conventional semen analysis

Semen analysis was carried out in accordance with the 5th Edition of WHO 2010 guidelines [18]. Semen samples were collected by masturbation into a sterile container after 2–6 days of sexual abstinence. All samples were left to liquefy at 37 °C and then analyzed by an ordinary light microscope (X400) for sperm concentration (106 /ml), total sperm motility (%), progressive motility (%), and morphology (% abnormal forms). For each semen sample, an aliquot of the whole semen was recovered and centrifuged at 3,000 g for 15 min to obtain SP. SP was immediately allocated into labelled and sterile tubes, stored at − 20 °C for subsequent assays of visfatin and vaspin.

#### Assessment of sperm DNA integrity using the SCD assay

The sperm chromatin dispersion (SCD) test is based on the idea that sperm with fragmented DNA cannot form the characteristic halo of dispersed DNA loops seen in sperm without fragmentation [19]. The study used a commercial kit called the Halosperm DNA kit (Halotech, Madrid, Spain). Sperm samples were mixed with melted agarose, pipetted onto a slide, covered with a coverslip, and kept at 4 °C. The samples were then processed with denaturation, lysis, washing, and dehydration steps. After staining, they were examined under a microscope. 200 sperms per sample were evaluated at × 400 magnification by two experienced lab personnel to minimize any potential bias.

### SDFI calculation

The SDFI was calculated as the ratio of the number of sperms with fragmented DNA to the number of all analyzed sperms. A SDFI value of < 30% was considered normal, according to the manufacturer’s guidance. However, SDFI is considered normal when it is ≤ 15 [20]. While it is considerd average, when it is > 15% to < 30%, and it is considerd high, when it is ≥ 30% [20].

#### SP visfatin level measurement

The SP visfatin levels were measured in ng/ml using a Sandwich ELISA kit (CAT: E0025Hu, Bioassay Technology Laboratory, Korain Biotech Co., Ltd., Shanghai, China) following the manufacturer’s guidance. The detection range of SP visfatin was 0.5–100 ng/ml; the inter-assay coefficient of variation (CV) was 6.4%; and the sensitivity was 0.23 ng/ml.

#### SP vaspin level measurement

The SP vaspin levels were measured in ng/ml using a commercial kit (Human Vaspin ELISA Kit, CAT: ELK2012) from ELK Bio-Technology Laboratory, USA. The kit has a sensitivity of 0.01 ng/ml and and intra-assay CV < 8% and an inter-assay CV < 10%.

An ELISA device (DAS Plate Reader, SN 2006, Italy) was utilized to to quantify the concentrations of SP visfatin, and vaspin.

### Statistical methods

Data were analyzed using SPSS version 28 (IBM Corp., Armonk, NY, USA), with quantitative variables presented as mean ± SD and categorical variables as frequencies or percentages. Comparisons between groups were done using analysis of variance (ANOVA) with a post hoc test for normally distributed quantitative variables. For comparing categorical data, χ^2^ test was performed. The exact test was used instead when the expected frequency was less than 5. Correlations between quantitative variables were done using the Pearson correlation coefficient. The receiver operating characteristic (ROC) curve was constructed to detect the best cut-off value of significant parameters for the detection of DNA fragmentation. Linear regression analysis was done to detect independent predictors of abnormal DNA fragmentation. *P*-values < 0.05 were considered statistically significant.

### Sample size calculation

The provided formula, *n* = Z^2^ x PQ/d^2^, was used to calculate the sample size. In this equation, n represented the required sample size, Z represented the confidence level at 95% (with a standard value of 1.96), P represented the estimated prevalence of high SDF, Q was equal to 1 minus P, and d represented the margin of error (0.05 in this case). The exact prevalence of high SDF was not known, but based on existing literature, it was believed to be less than 1% of the population [21]. By substituting the values into the equation, we obtained the result: n = (1.96)^2^ * (0.01) * (1–0.01) / (0.05)^2^, which was approximately = 30.

## Results

The current study included ninety participants, who had an average age of 40.24 ± 7.9 years. The participants had a mean BMI of 27.9 ± 4.5. The mean SDFI was determined to be 18.41 ± 15.38, while the mean levels of SP visfatin and vaspin were 20.5 ± 7.51 ng/ml and 0.72 ± 0.38 ng/ml, respectively. The sociodemographic characteristics of all participants are detailed in (Table 1). A comparison of demographic and clinical characteristics in relation to DNA integrity revealed no statistically significant difference between the three groups (Table 2). Conversely, a comparison of SP vaspin and SP visfatin levels revealed substantial variations between groups with good, medium, and poor DNA integrity (Table 2). The group with good DNA integrity exhibited significantly higher levels of sperm concentration and progressive motility compared to the remaining groups (Table 2). On the other hand, both groups with good and medium DNA integrity exhibited significantly higher total motile sperm count (TMC) and sperm motility in comparison to the group with poor DNA integrity (Table 2). Additionally, the group with good DNA integrity demonstrated a significantly lower percentage of abnormal forms compared to the group with compromised DNA integrity (Table 2). Significant negative correlations were noticed between SDFI and numerous semen parameters, such as sperm concentration, TMC, total motility, progressive motility, and vitality (Table 3). In a similar trend, a significant negative correlation was observed between SDFI and SP vaspin (Table 3). On the other hand, a significant positive correlation was found between SDFI and the percentage of abnormal forms (Table 3). Regarding the relationship between demographic characteristics and SP vaspin, a statistically significant negative correlation was identified with age (*r* = -0.305, *P* = 0.006) and infertility duration (*r* = -0.263, *P* = 0.019) (Table 3).

**Table 1:** Socio-demographic and clinical and laboratory characteristics of the participants

Variable		Mean	SD	Minimum	Maximum
Age (years)		40.24	± 7.91	23	63
Infertility duration (years)		7.42	± 5.73	1	29
Height (cm)		174.39	± 7.50	160	190
Weight (Kg)		84.77	± 14.25	55	105
BMI (kg/m^2^)		27.90	± 4.49	17.6	31
Semen volume (ml)		3.46	± 1.67	0.4	12
Semen PH		7.52	± 0.062	7.5	7.8
Sperm concentration (10^6^/ ml)		53.50	± 43.85	5.5	190
TMC count (10^6^) / ml		24.01	± 13.79	1	107
TMC count (10^6^)/ ejaculate		82.73	± 64.74	2	643
Motility (%)		38.86	± 17.65	5	65
Progressive motility (%)		8.76	± 4.64	1	25
Vitality (%)		55.57	± 16.25	20	78
Abnormal forms (%)		94.33	± 3.78	82	100
Leukocyte count (10^6^ / ml)		0.2	± 0.06	0	40
SDFI (%)		18.41	± 15.38	2	80
Seminal plasma vaspin (ng/ml)		0.72	± 0.38	0.15	1.78
Seminal plasma visfatin (ng/ml)		20.50	± 7.51	10	57
		number		percentage	
Occupation	Risky	15		16.7%	
	Non-risky	75		83.3%	
Special habits	Smoker	12		13.3%	
	Non-smoker	78		86.7%	
Varicocele	No	42		46.7%	
	Left	14		15.5%	
	Bilateral	34		37.8%	
DNA integrity	Normal (SDFI ≤ 15%)	33		36.7%	
	Medium (15% < SDFI < 30%)	29		32.2%	
	Abnormal (SDFI ≥ 30%)	28		31.1%	

N.B. ± SD (standard deviation) or frequency (%)

*BMI* Body mass index, *SDFI* Sperm DNA fragmentation index, *TMC* Total motile count

**Table 2:** Socio-demographic and clinical characteristics and sperm parameters and seminal plasma visfatin and vaspin levels in relation to DNA integrity

		Normal SDFI group [*n* = 33]	Medium SDFI group [*n* = 29]	Abnormal SDFI group [*n* = 28]	*P* value
Age (years)		39.58 ± 6.18	39.24 ± 7.20	43.24 ± 11.22	0.211
Infertility duration (years)		7.55 ± 5.88	7.07 ± 5.32	7.76 ± 6.38	0.913
Occupation	Risky	4(12.1%)	5(17.2%)	6(21.4%)	0.080
	non risky	29(87.9%)	24(82.8%)	22(78.6%)	
Special habits	Smoker	3(9.1%)	4(13.8%)	5(17.9%)	0.107
	Non-smoker	30 (90.9%)	25(86.2%)	23(82.1%)	
Height (cm)		175.09 ± 7.12	173.69 ± 7.85	174.24 ± 7.95	0.765
Weight (Kg)		87.24 ± 15.94	82.83 ± 13.53	83.29 ± 11.80	0.430
BMI (Kg/m^2^)		28.39 ± 4.50	27.54 ± 4.64	27.58 ± 4.39	0.720
Varicocele	No	15(45.4%)	14(48.3%)	13(46.4%)	0.568
	Left	6(18.2%)	4(13.8%)	4(14.3%)	
	Bilateral	12(36.4%)	11(37.9%)	11(39.3%)	
Sperm concentration (10^6^/ ml)		62.65 ± 45.19	54.78 ± 46.75	33.56 ± 29.44	0.042^a^
TMC count (10^6^) / ml		30.93 ± 19.35	25.49 ± 11.13	10.42 ± 7.06	< 0.001^b^
TMC count (10^6^)/ ejaculate		146.63 ± 116.47	65.99 ± 42.92	29.37 ± 19.35	< 0.001^b^
Motility (%)		44.24 ± 15.65	43.21 ± 13.59	21.00 ± 16.50	< 0.001^b^
Progressive motility (%)		9.27 ± 6.56	5.48 ± 4.42	4.86 ± 3.29	0.023^a^
Vitality (%)		61.56 ± 12.62	58.17 ± 20.07	50.93 ± 16.21	0.282
Abnormal forms (%)		91.97 ± 4.39	95.10 ± 2.86	95.65 ± 3.18	0.021^a^
Leukocyte count (10^6^ / ml)		0.28 ± 0.04	0.26 ± 0.02	0.22 ± 0.03	0.548
Seminal plasma vaspin (ng/ml)		0.87 ± 0.39	0.68 ± 0.39	0.51 ± 0.23	0.005^a^
Seminal plasma visfatin (ng/ml)		22.45 ± 5.59	19.19 ± 10.05	18.96 ± 4.75	0.004^c^

Values are mean ± SD or frequency (%)

N.B. *BMI* Body mass index, *SDFI* Sperm DNA fragmentation index, *TMC* Total motile count

^a^Group I is significantly different from group 3

^b^Group III is significantly different from group 1 and group 2

^c^Group I is significantly different from group 2 and group 3

**Table 3:** Correlations of SDFI and seminal plasma vaspin and visfatin with clinical characteristics and sperm parameters

	SDFI (*n* = 90)		Seminal plasma Vaspin (*n* = 90)		Seminal plasma Visfatin (*n* = 90)	
	*r*	*P* value	*r*	*P* value	*r*	*P* value
Age (years)	0.127	0.264	-0.305	0.006	0.126	0.268
Infertility duration (years)	0.043	0.707	-0.263	0.019	0.139	0.221
Height (cm)	-0.059	0.606	0.106	0.351	0.100	0.378
Weight (kg)	-0.075	0.513	-0.010	0.929	0.095	0.407
BMI (kg/m^2^)	-0.026	0.819	-0.083	0.466	0.041	0.722
Sperm concentration (10^6^/ ml)	-0.282	0.012	0.098	0.392	-0.081	0.480
TMC count (10^6^) / ml	-0.365	0.001	0.094	0.412	-0.060	0.602
TMC count (10^6^)/ ejaculate	-0.319	0.004	0.049	0.668	0.036	0.751
Motility (%)	-0.578	< 0.001	0.219	0.052	0.050	0.659
Progressive motility (%)	-0.319	0.004	0.103	0.366	0.118	0.301
Vitality (%)	-0.405	0.027	0.033	0.861	0.041	0.831
Abnormal forms (%)	0.227	0.044	-0.073	0.524	-0.239	0.034
Leukocyte count (10^6^ / ml)	-0.075	0.510	0.013	0.911	0.195	0.085
SDFI	-	-	-0.335	0.003	-0.166	0.143
Seminal plasma Vaspin(ng/ml)	-0.335	0.003	-	-	-0.021	0.855
Seminal plasma Visfatin(ng/ml)	-0.166	0.143	-0.021	0.855	-	-

N.B. *BMI* Body mass index, *SDFI* Sperm DNA fragmentation index, *TMC* Total motile count

**Fig. 1:**
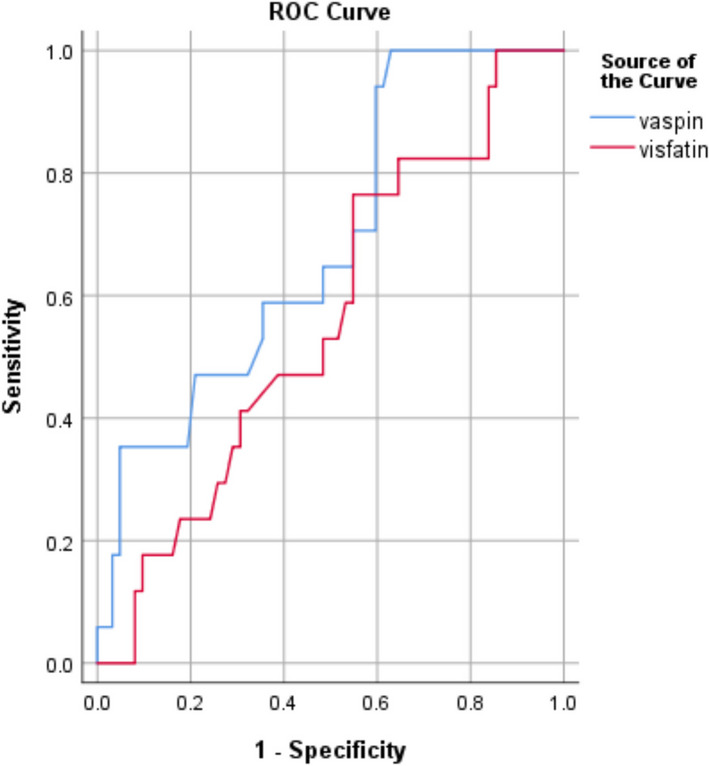
ROC curve for prediction of DNA fragmentation ≥ 30% using vaspin and visfatin

Additionally, a statistically significant negative correlation was identified between SP visfatin and the percentage of abnormal forms (*r* = -0.239, *P* = 0.034) (Table 3). It was observed that patients with risky occupations exhibited significantly higher vaspin levels (0.93 ± 0.45 ng/ml) compared to those with non-risky occupations (0.68 ± 0.36 ng/ml, *p* = 0.031) (Table 4). Nevertheless, the statistical analysis yielded no significant influence (*p* > 0.05) of risky occupations, varicocele, or smoking on the levels of SP visfatin (Table 4). The ROC curve for predicting poor DNA integrity (SDFI ≥ 30%) revealed fair discriminative power for SP vaspin, with a cutoff value of < 0.55 ng/ml. It demonstrated a sensitivity of 58.8% and a specificity of 64.5% (AUC 0.685, *p* = 0.008) (Fig. 1). Meanwhile, SP visfatin had little discriminative power (AUC 0.562, *p* = 0.408) (Fig. 1). Finally, the results of a linear regression analysis indicated that sperm motility and SP vaspin were significant independent predictors of poor DNA integrity (SDFI ≥ 30%) (Table 5). The analysis was done with a 95% confidence interval (CI) and showed upper and lower bounds of -0.302 and -0.623, and -1.362 and -16.101, respectively (Table 5). The *p*-values were less than 0.001 for the first predictor and 0.021 for the second predictor (Table 5).

**Table 4:** Levels of seminal plasma vaspin and visfatin in relation to occupation and specific habits and varicocele

		Seminal plasma Vaspin (ng/ml)		*P* value	Seminal plasma visfatin (ng/ml)		*P* value
		Mean	SD		Mean	Sd	
Occupation	Risky	0.93	± 0.45	0.031	18.69	± 5.84	0.5
	Non risky	0.68	± 0.36		20.89	± 7.81	
Special habits	Smoker	0.80	± 0.41	0.432	19.91	± 5.16	0.956
	Nonsmoker	0.71	± 0.38		20.61	± 7.88	
Varicocele	No	0.61	± 0.27	0.148	21.67	± 9.53	0.761
	Left	0.85	± 0.43		20.19	± 5.71	
	Bilateral	0.77	± 0.44		19.57	± 5.98	

Values are mean ± SD

**Table 5:** Multivariate Linear regression to detect independent predictors of poor DNA integrity (SDFI ≥ 30%)

Model		Unstandardized Coefficients		Standardized Coefficients	T	*P* value	95.0% Confidence Interval for B	
		B	Std. Error	Beta			Lower Bound	Upper Bound
SDFI ≥ 30%	(Constant)	42.669	3.898		10.946	< 0.001	34.905	50.432
	Motility (%)	-0.462	0.081	-0.530	-5.728	< 0.001	-0.623	-0.302
	Seminal plasma vaspin (ng/ml)	-8.732	3.700	-0.218	-2.360	0.021	-16.101	-1.362

## Discussion

**Fig. 2:**
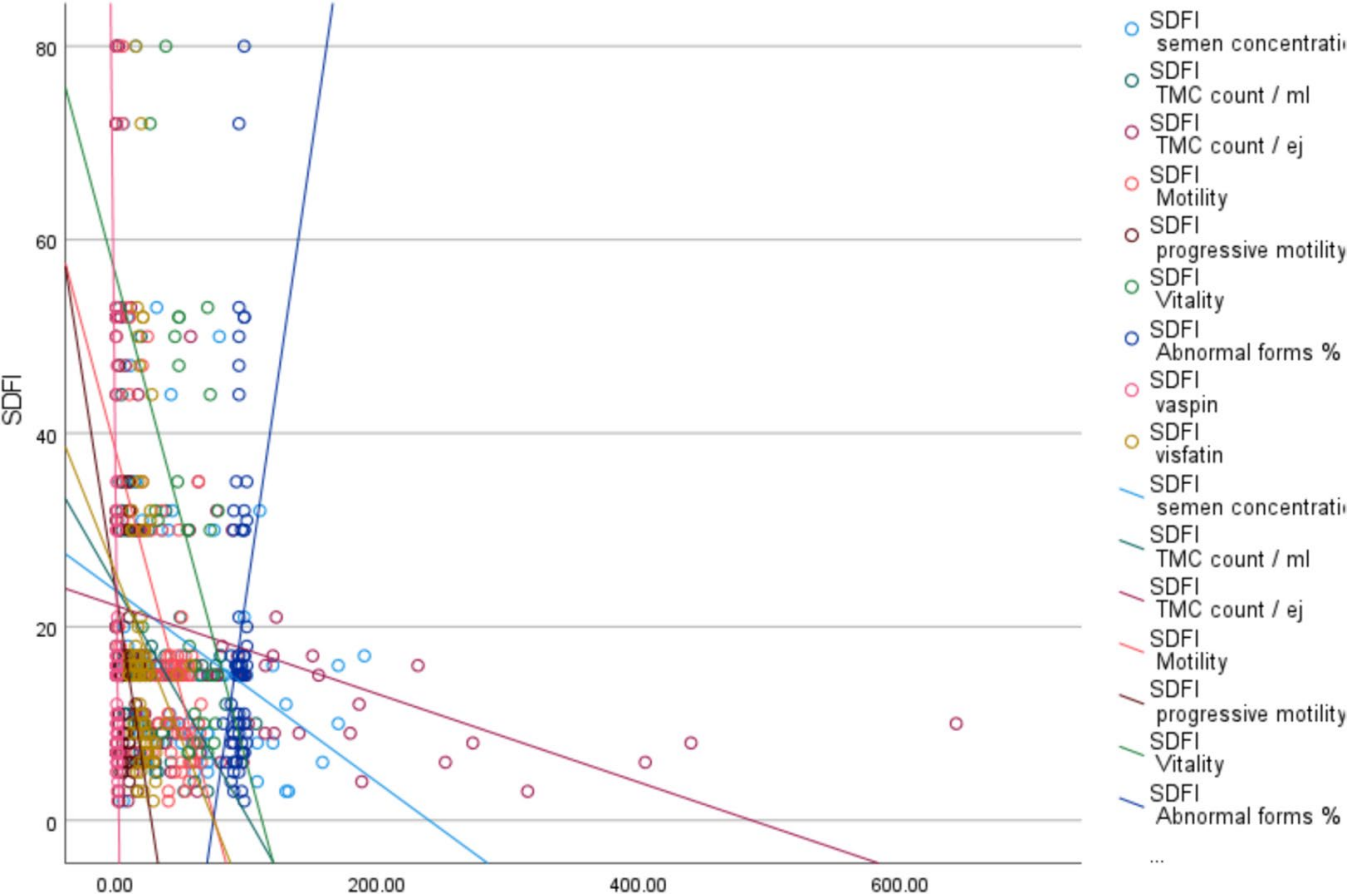
Shows the correlation between all studied parameters and sperm DNA fragmentation

The current study had shown significant variations in sperm concentration, sperm motility, progressive motility, and TMC between the group exhibiting favorable DNA integrity and the group displaying poor DNA integrity. In a similar trend, Zidi-Jrah et al. (2016) identified positive associations between good DNA integrity and semen parameters [22]. Moreover, the proportion of abnormal forms observed in the present study exhibited a significant decrease in the group with good DNA integrity when compared to the group with poor DNA integrity (Fig. 2).

Consistently, Le et al. (2019) revealed a positive correlation between the SDFI and abnormal head morphology, while a weak negative correlation was found with progressive motility [23]. The current study highlighted the importance of sperm motility as a significant indicator of DNA integrity. There is an overwhelming debate among researchers about the relationship between SDFI and semen parameters [3]. Some studies had shown a correlation between SDFI and sperm concentration, motility, and morphology, particularly in cases of abnormal DNA [24, 25]. Nevertheless, other studies had not found a significant association between SDFI and conventional semen parameters [2, 26]. Additionally, some studies had reported conflicting results, with SDF only showing a correlation with a single parameter like morphology [27] or motility [26]. The discrepancies in findings might be due to variations in techniques for assessing DNA integrity, criteria for analyzing semen parameters, quality control measures during testing [28]. Also, it might be attributed to the lack of consistency in selecting criteria for the studied population [29]. The present study displayed a significant negative correlation between SDFI and sperm concentration, motility, progressive motility, and vitality, as well as a positive correlation with abnormal form percentage. In the same context, Choucair et al. (2016) revealed a higher SDF in infertile individuals compared to controls, with SDF correlating with alterations in sperm parameters [30]. This finding was also reported by Belloc et al. (2014) [26]. Regarding demographic and clinical characteristics in relation to DNA integrity did not reveal statistically significant differences across all three groups including smoking. However, the current study demonstrated that occupations involving potential hazards could have an impact on SP vaspin levels. Conversely, Choucair et al. (2016) had suggested an association between SDF and tobacco use and environmental factors [30].

Furthermore, aging, risky occupations, smoking, and obesity had been linked to SDF development in various studies [31–34]. In contrast, Anagnostopoulou and colleagues (2022) found no correlation between environmental factors and SDF [35]. The findings obtained in the present study might be attributed to the fact that there were only a limited number of participants involved. Moreover, a limited percentage of the individuals included in the study were smokers or had hazardous occupations. Additionally, a minority of the participants were morbidly obese or advanced in age. Visfatin had been found to have a potential impact on male fertility in animal models, with higher levels of visfatin associated with poorer sperm quality in rats with diabetes and obesity [13]. However, conflicting results had been reported regarding the relationship between visfatin and sperm parameters in different studies [36, 37]. In our study, there was no significant relationship between SP visfatin and SDFI. Nevertheless, SP visfatin levels had been shown to be significantly higher in men with good sperm DNA integrity compared to those with medium or poor integrity. The aforementioned fact could be explained by the fact that It has been shown that DNA damage induced by high levels of ROS can activate the poly(ADP-ribose) polymerase (PARP) system to trigger DNA repair [38]. However, the activity of the PARP-DNA repair response depends on NAD as its substrate [39]. In this case, a low level of visfatin, which plays a regulatory role in NAD biosynthesis, could affect PARPs as NAD-dependent proteins and impair DNA repair [15]. Instead, Anagnostopoulou et al. (2022) did not report differences in SP visfatin levels between fertile and infertile male participants [35]. The present study showed that SP visfatin levels were negatively correlated with the percentage of abnormal forms.

In contrast, Anagnostopoulou et al. (2022) observed a significant negative correlation between SP visfatin levels and sperm count and concentration [35]. Moreover, a recent meta-analysis revealed that males with reduced DNA integrity significantly exhibited lower levels of progressive and total motility compared to individuals with normal DNA integrity [40]. The findings of the present study provided valuable insights into the relationship between sperm DNA integrity and SP vaspin levels. Our results indicated that individuals with good SDFI notably exhibited higher levels of SP vaspin compared to those with poor SDFI. In a similar trend, Thomas et al. (2013) observed a positive correlation between SP vaspin and SDF [36]. This suggested that SP vaspin might play a role in maintaining sperm DNA integrity. Furthermore, a statistically significant negative correlation was observed between SP vaspin and SDFI. This finding expands our understanding of the factors influencing sperm DNA integrity and provids a foundation for further research in this area. It also suggests that SP vaspin may serve as a potential biomarker for assessing sperm DNA quality and could potentially be targeted for therapeutic interventions aimed at improving male fertility. However, future research is needed to fully elucidate the underlying mechanisms and clinical implications of this relationship.

### Limits of the study

Remarkably, it should be mentioned that the study was mainly limited by the inability to utilize the 6th Edition of WHO manual for semen analysis [41]. The 6th Edition was proposed to improve semen analysis procedures by including structured steps and a methodological sequence for test performance [41]. Furthermore, this manual presented new sperm tests for the assessment of SDF and seminal OS, while deleting historical tests like human cervical mucus [41].

Moreover, the 6th Edition was proposed to address the cons of the 5th Edition related to the demographic under-representation of some geographical regions. Nevertheless, it should be mentioned that the 5th Edition facilitated the standardization of semen analysis procedures through a structured step-by-step approach to various standard and extended semen tests [18]. Additionally, it contained a comprehensive part on cryopreservation, which is essential for fertility preservation and assisted reproductive techniques (ART) together with sperm processing for testicular and epididymal sperm, as it’s guidelines promoted better handling across the clinical andrology and ART laboratories [18]. Consistently, there are several concerns about the 6th Edition that needs further clarification for andrologists [42]. These weaknesses include underrepsentation of fertile people from some geographical locations, lack of decision limits to replace the 5th percentiles of the basic semen parameters, indications and criteria as well as cutoff / threshold values for proper analysis of sperm DNA fragementation testing and finally devaluing the role of seminal oxidative stress in male infertility [41]. Additionally, the small sample size and the exclusion of morbidly obese and elderly patients should be added as a limitation. Furthermore, SDFI was evaluated by SCD test only that can be added as a further limitation. However, Liffner et al. (2019) stated that sperm chromatin structure analysis and SCD assay produce similar results as regard SDFI [43].

## Conclusions

SP Level of vaspin had shown promise as potential biomarkers for sperm DNA integrity. However, vaspin appeared to have greater specificity than visfatin in this point. Future studies are required to validate these findings, evaluate the role of SP vaspin in maintaining sperm DNA integrity, and investigate the potential relationship between SP adipocytokines and other clinical-demographic variables.

## Data Availability

The data that support the fndings of this study are available from the corresponding author upon reasonable request.

## Abbreviations

BMI: Body mass index
CV: Coefficient of variation
NAD: Nicotinamide adenine dinucleotide
NADH: Nicotinamide adenine dinucleotide + hydrogen
ROC: Receiver operating characteristic
SCD: Sperm chromatin dispersion
SDF: Sperm DNA fragmentation
SDFI: Sperm DNA fragmentation index
SP: Seminal plasma
PARP: Poly(ADP-ribose) polymerase
TMC: Total motile count
ART: Assisted reproductive techniques
